# Quality of life after spinal cord injury: a qualitative interview-based study

**DOI:** 10.1038/s41394-026-00735-3

**Published:** 2026-04-25

**Authors:** Vasilios Stenimahitis, Amina Guenna Holmgren, Maria Gharios, Victor Gabriel El-Hajj, Victor E. Staartjes, Claes Hultling, Adrian Elmi-Terander, Erik Edström

**Affiliations:** 1 Department of Clinical Neuroscience, Karolinska Institutet, Stockholm, Sweden; 2 Department of Rehabilitation, Furuhöjden Rehab Hospital, Täby, Sweden; 3 Department of Neurobiology, Care Sciences and Society, Karolinska Institutet, Stockholm, Sweden; 4 Machine Intelligence in Clinical Neuroscience & Microsurgical Neuroanatomy (MICN) Laboratory, Department of Neurosurgery, Clinical Neuroscience Center, University Hospital Zurich, University of Zurich, Zurich, Switzerland; 5 Capio Spine Center Stockholm, Löwenströmska Hospital, Upplands-Väsby, Sweden; 6 Department of Medical Sciences, Örebro University, Örebro, Sweden; 7 Department of Surgical Sciences, Uppsala University, Uppsala, Sweden

**Keywords:** Outcomes research, Diseases of the nervous system

## Abstract

**Study design:**

Qualitative, descriptive study of audio recorded, transcribed and analyzed data, based on individual semi-structured interviews using an interview guide.

**Objectives:**

To describe the subjective experience and quality of life (QoL) of individuals with spinal cord injury (SCI) and identify elements of importance in determining QoL.

**Setting:**

Swedish outpatient clinic.

**Methods:**

Individuals with SCI were recruited from a specialized SCI outpatient clinic, with eligibility limited to those who had attended at least one prior clinical follow-up. No interventions were applied. Data were collected through qualitative interviews, and the primary outcome measure was the identification of key themes influencing QoL following SCI, as perceived and reported by participants.

**Results:**

The interviews identified several key factors with perceived impact on the subjects’ QoL including difficulties in managing everyday life, the desire to live an independent life, the significance of community and a sense of belonging, current life situation in relation to others and past experiences, and dealing with physical problems, and in particular pain, related to SCI.

**Conclusion:**

Participants highlighted independence, social connections, and managing SCI- related physical issues as crucial for their quality of life. They emphasized the community’s role in helping individuals with SCI, live meaningful lives and pursue personal goals and aspirations.

## Introduction

Spinal cord injury (SCI) is a life-altering condition resulting in varying degrees of functional impairment and consequently bringing about a negative impact on the individual with SCI [1, 2]. Individuals with SCI may experience sensory-motor deficits, loss of bladder or bowel control, cardiovascular compromise, pressure ulcers and pain, along with an increased risk for anxiety and depression, resulting in the loss of personal independence [3–10]. Quality of life (QoL) measures are important tools in assessing the broad impact of SCI on the individual [5, 7, 8].

Currently, there is no consensus on the definition of QoL [5, 7, 11, 12]. The World Health Organization (WHO) provides the following definition: “… an individual’s perception of their position in life in the context of the culture and value systems in which they live and in relation to their goals, expectations, standards and concerns” [13]. Within the framework of the WHO definition, QoL is a multidimensional concept with different domains, integrating parameters such as physical and psychological health, social relationships, personal beliefs and level of autonomy [13].

There are several reports on the difficulties of deciphering QoL data for clinical application, and revisions have been made to standardize the assessment tools [14, 15]. In 1998, the WHO Quality of Life Group, introduced an abbreviated version of QoL assessment, mainly focusing on four domains; physical and psychological health, social relationships and environment [16]. A common ground regarding the standardization of QoL measurement in individuals with SCI was achieved through the presentation of the International Spinal Cord Injury QoL Basic Data Set (SCI-QoL-BDS) [11], which is a form consisting of questions covering the three most significant subjective domains of QoL in SCI: overall well-being, physical and psychological health. The SCI-QoL-BDS has emerged as a valid and useful tool for collecting QoL information in both clinical and research settings, and may assist in the identifying of possible adjustment difficulties among individuals with SCI [17]. In an international validation study by Rohn et al. in 2022, the identification of a fourth domain regarding social life was endorsed [15], which eventually prompted its addition as a parameter in the second version of the questionnaire [18].

Significant improvements in healthcare and rehabilitation during the past decades have led to a reduction in the mortality after SCI [8, 19]. To ensure reduced mortality also leads to meaningful lives for individuals with SCI, this study used semi-structured interviews and a qualitative design to explore their subjective experiences, addressing a gap in existing research.

## Methods

A qualitative descriptive study design was used to explore patient experience of QoL after SCI [20, 21]. Data were collected using semi-structured individual interviews, conducted with an interview guide [22].

Participants included in the study were individuals with SCI who had completed at least one follow-up clinical assessment. Participants were recruited in Stockholm, Sweden through the region’s only specialized SCI care Outpatient Clinic, which serves an area of 2.3 million inhabitants.

All participants were ≥18 years old, Swedish-speaking, and without severe cognitive impairment. Study information was emailed to potential participants. Efforts were made to ensure diversity in age, sex, and injury severity. Interested individuals received detailed information and were scheduled for interviews.

Using an interview guide, semi-structured individual interviews were performed. The interview guide consisted of the following questions: (1) How satisfied are you with your physical well-being during the last two weeks? (2) How satisfied are you with your psychological wellbeing during the last two weeks? (3) How satisfied are you with your social situation, professionally or privately during the last two weeks? (4) How satisfied are you with your environment and your situation at home during the last two weeks? (5) How satisfied are you with your life in total when you think about your personal circumstances? (6) What does the term “quality of life” mean to you? Questions 1 to 5 were presented as visual analogue scales ranging from zero (worst) to ten (best). The numerical responses are presented in Table 2.

After responding to Questions 1 to 5, additional probing questions were asked to obtain more detailed explanation and gain deeper insights on each specific topic. The participants were also asked to provide an example from everyday life that illustrated their perception of QoL. The quantitative ratings provided an understanding which guided the follow-up questions and helped identify the key areas of importance to each participant. Thematic categories were then generated from the analysis of the narrative responses. The interviews were conducted in Swedish, either in person or with the use of a videotelephony software program. The audio was recorded, and transcribed verbatim. The Swedish transcripts were then translated into English by bilingual members of the research team. Back-translation was performed for selected text segments requiring deeper interpretation to ensure semantic preservation and conceptual equivalence. Additional baseline data were collected from participants, including age, sex, level of injury, completeness of injury (complete vs. incomplete), time since injury, and cause of SCI. Level of injury and completeness were reported in accordance with the ASIA/ISCoS International Standards for Neurological Classification of Spinal Cord Injury (ISNCSCI). For age and time since injury, data were grouped into 10-year intervals to ensure participant anonymity (Table 1).

**Table 1:** Participant characteristics.

Participant	Age	Sex	Injury Level	Completeness	Years since injury	Cause of SCI
1	51-60	F	Tetraplegia	Complete	31-40	Trauma
2	41-50	M	Tetraplegia	Complete	21-30	Trauma
3	41-50	M	Paraplegia	Complete	11-20	Trauma
4	61-70	F	Paraplegia	Complete	31-40	Trauma
5	71-80	F	Paraplegia	Incomplete	31-40	Infectious
6	71-80	F	Paraplegia	Incomplete	41-50	Trauma
7	51-60	M	Tetraplegia	Complete	31-40	Trauma
8	41-50	M	Tetraplegia	Complete	11-20	Trauma
9	31-40	M	Tetraplegia	Incomplete	11-20	Trauma
10	51-60	M	Tetraplegia	Complete	31-40	Trauma
11	11-20	M	Tetraplegia	Complete	1-10	Trauma
12	50-60	M	Paraplegia	Complete	31-40	Trauma
13	31-40	M	Paraplegia	Complete	21-30	Trauma

### Data analysis

The interviews were analyzed using inductive manifest qualitative content analysis [23]. In summary, transcripts were repeatedly reviewed to identify meaning units aligned with the study aim. These were coded, compared for patterns, and grouped into categories through iterative analysis. Two authors coded independently, and consensus was reached through discussion.

### Ethical considerations

The study was conducted in accordance with the tenets of the Declaration of Helsinki and was approved by the Swedish Ethical Review Authority (Dnr 2024-03243-01). Written informed consent or audio-video recorded verbal informed consent was obtained from all study participants prior to enrollment.

## Results

**Fig. 1:**
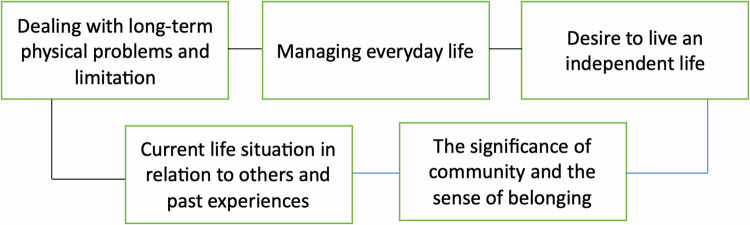
Illustration of the five central thematic categories identified through analysis of the interview transcripts. The figure displays the five main themes emerging from the qualitative analysis, representing the core patterns and concepts identified across participant narratives.

Each interview lasted approximately 25-35 min, with an average duration of 30 min. The participants’ characteristics were self-reported during the interviews (Table 1). Numerical responses on Questions 1-5; addressing physical wellbeing, psychological wellbeing, social situation, environment and home situation, and life in total, were summarized (Table 2). The analysis of interview transcripts revealed five central thematic categories that participants commonly emphasized: (1) Dealing with long-term physical problem and limitations, (2) Managing everyday life, (3) Desire to live an independent life, (4) Current life situation in relation to others and past experiences and (5) The significance of community and the sense of belonging (Fig. 1).

**Table 2:** Participants numerical responses to Questions 1-5 (scale 0-10).

Participant	Question 1	Question 2	Question 3	Question 4	Question 5
1	8	10	10	10	8.5
2	6	7.5	7.5	7.5	8
3	4	6	7	9	7
4	7	8	8	9	9
5	6	10	10	10	10
6	3	6	8	8	9
7	7.5	8	9	9	8
8	7	7	9	10	9
9	7	8	9	7	7.5
10	5	7	9	7	8
11	7	7	8.5	8	8
12	3	6	5	8	6
13	6.5	9	9	9	9
Mean score	5.92	7.65	8.38	8.57	8.23

(1) How satisfied are you with your physical well-being during the last two weeks?

(2) How satisfied are you with your psychological wellbeing during the last two weeks?

(3) How satisfied are you with your social situation, professionally or privately during the last two weeks?

(4) How satisfied are you with your environment and your situation at home during the last two weeks?

(5) How satisfied are you with your life in total when you think about your personal circumstances?

### 1 - Dealing with long-term physical problems and limitations

Physical problems and limitations related to SCI were presented as fundamental elements in the participants’ perceptions of QoL. Physical and functional limitations had a negative impact on the individuals’ psychological well-being. Anxiety and uncertainty about the prospect of surgical treatment for related health problems - such as device implantation for spasticity or revision of internal fixation material - were reported, along with concerns about potential complications. In this context, the importance of maintaining overall health was particularly emphasized. Participants reported that successful surgical interventions had a positive impact on QoL, while deterioration of overall health and development of additional medical conditions elicited negative emotional responses. SCI-related long-term complications, such as pain, bladder and bowel dysfunction, spasticity and impact on sexuality, appeared frequently across the participants’ responses with a negative impact on the overall perception of QoL and overlapped with other areas such as social life, ability to stay active and participate in sports.

*“By eliminating or reducing the physical problems, the psychological wellbeing would improve.” (Participant 9)*

The use of coping strategies such as pushing aside thoughts related to physical limitation, was often mentioned. Similarly, the negative impact of neuropathic pain on QoL was a recurrent theme. Reflections on the administration of advanced treatments for pain and the side-effects profile of various medications were also frequently brought up across the interviews.

*“We all become older, we have cramps, some pain, some arthritis and so on…, but sitting in a wheelchair leaves then so much less margin for every little shortcoming.” (Participant 12)*

### 2 - Managing everyday life

The participants expressed dissatisfaction with the limited availability of formal assistance in daily care and limited accessibility of public spaces and workplaces. Additionally, participants reported insufficient financial support to afford required medical aids including adaptations to their vehicles and homes. These feelings translated into circumstantial anxiety and frustration, highlighting in this context the particular importance of financial security. Concern and uncertainty about the availability of additional assistance and ease of access to health care in the event of future medical problems or complications were reported. In that respect, participants also reflected on the complicated administrative and bureaucratic profile of the current health care system.

*“I used to rely on my husband, but he can’t do it anymore. If his condition worsens, I worry about who will help me; and that uncertainty causes anxiety that takes a toll on my mental health.” (Participant 4)*

High quality of provided care and access to formal support including necessary medical devices were deemed as important factors for QoL. Moreover, satisfaction with the working environment was deemed as an essential QoL parameter. In contrast, a heavy workload was recognized as a significant challenge with negative impact on QoL. The need of additional informal assistance from family members or relatives was also described as unavoidable.

*“I have a fantastic family that supports as needed… I am satisfied with formal personal assistance, but some things could move a little faster” (Participant 11)*

### 3 - Desire to live an independent life

Autonomy, including self-reliance and performing daily activities, was key to QoL. Participants described the ongoing struggle with SCI-related issues, worsened by aging, which reduced independence and triggered negative emotions. Self-efficacy in tasks like driving and managing assistance needs positively influenced QoL. Empowerment through decision-making and personal responsibility was frequently highlighted as vital.

*“Quality of life is, in some way, having a situation where you feel that you can achieve the things you want or dream about. Maybe not all dreams, but at least the realistic ones.” (Participant 10)*

Reflections on the state of being able to engage in similar activities performed by others in the society were explored. Work-related aspects, such as the prospect to work remotely from home and with a flexible schedule to allow time for traveling or recreation and sports were touched upon. Within this frame of reference, reflections on the interrelationship between high working performance accompanied by satisfaction with one’s work were associated with QoL, however the downside of a too intense workload on physical performance was underlined.

*“The most important thing is to feel that it’s not impossible to do things… that I, you, and everyone else can do them, and that there should be information about it and all that, about how to do it.” (Participant 11)*

### 4 - Current life situation in relation to others and past experiences

Psychological responses and reflections, on ability to perform tasks and in relation to philosophy about life after SCI were explored oftentimes by the participants. Participants expressed appreciation for maintaining intact cognitive function and noted that cognitive impairment would be more detrimental than the physical impairment associated with SCI. Recalibration of life expectations and exploration of different career paths were also expressed.

*“Quality of life is important, I feel that I have it, I feel good with what I have, and with what I have around me and with what I do” (Participant 13)*

Participants reflected that they often try to look at their SCI from a different perspective; by comparing their physical disability to other individuals with more severe SCI or even other more debilitating medical conditions. Additionally, participants also felt that their weaknesses or limitations became more apparent when interacting with or compared to other people. In the context of physical limitations, participants expressed sadness and grief over the physical abilities they had lost and the activities they could no longer perform a result of their injury.

*“Sometimes you can feel a bit limited in your body… then I can miss going out for a run or a bike ride.” (Participant 1)*

Some of the coping strategies implemented by the participants included maintaining religious faith, participating in a group or congregation, and focusing on their academic interests.

### 5 - The significance of community and the sense of belonging

The meaningfulness of social factors, including immediate family, friendly relationships and professional contacts, in the participants’ perception of QoL was acknowledged consistently across the interviews.

*“Life is so much more than just the body and all that… I feel good and am doing well because of my family and loved ones. I can always call someone; I can always visit someone.” (Participant 1)*

Solid relationships with family members, friends and colleagues, were strongly associated with increased QoL. However, reflections on the loss of romantic relationships were also explored. Maintaining a good social life and spending quality time with family and friends – such as going out for dinner or traveling with them – had a strong positive impact on QoL.

*“We must have water, food and a social context to belong, you must be included in the community, you must belong…if you don’t, then you will feel bad” (Participant 12)*

The importance of feeling connected to and appreciated by others, was repeatedly emphasized. On the other hand, feelings of loneliness or disconnection from the community were identified as determinants of poor QoL.

*“The most important thing is to have a quality of life… to have a purpose, a context, a meaning. For me, it’s very important to have a job… to feel needed and appreciated.” (Participant 3)*

## Discussion

Everyday challenges for individuals with SCI can be overwhelming and emotionally taxing. Managing physical issues, daily activities, social connections, and community reintegration are consistently identified as key determinants of QoL in previous studies [24, 25].

The availability of appropriate personal assistance is essential for individuals with SCI, and its noteworthy importance for independence and participation in society cannot be overstated [26, 27]. Reflections regarding the significance of availability, or the lack thereof, of sufficient personal assistance were particularly underlined by the participants, highlighting the crucial role of personal assistance towards improving wellbeing and its impact on QoL. Within this framework, the financial strain associated with securing necessary assistive devices and necessary home modifications was revealed as a potential determinant of QoL, highlighting the need for secure allocation of appropriate financial resources by policymakers. In a qualitative study investigating factors that impact survivorship among individuals with SCI, Kayani et al. emphasized participants’ reported challenges related to the loss of independence after SCI, and the resulting negative self-perception [24]. In this study, participants expressed the same feelings, emphasizing the negative impact of losing independence.

The great importance of social factors and integration in society, on the perception of QoL in individuals with SCI is well recognized [28–31]. The involvement and participation in meaningful activities, both leisurely and professionally, promotes the process of re-establishment in the community [31]. Analogously, in this study the participants identified the beneficial effect of building a bond with the community through participation in its activities, focusing to the positive impact on subjective QoL of being valued within the community.

The potential effect of SCI as a possible stressor in both present and future intimate relationships has been explored in previous reviews, in addition to being penetrated by several of the participants [32–34]. The loss of independence in the aftermath of SCI and the extreme necessity for the development of coping strategies, alters significantly the dynamic of a relationship, where a spouse may undertake to role of a caregiver, and in turn may put an intimate partnership at risk [32]. Kreuter et al reported, in a study regarding partner relationships in individuals with traumatic brain injury and spinal cord injury, that the injury is not a substantial obstacle to the establishment of a partner relationship [33]. Conversely, Jeyathevan et al, in a qualitative analysis of interview data in individuals with SCI, reported on the experienced loss of intimate relationships and sex in the aftermath of SCI [34]. In our study, reflections regarding the loss of intimate relationships came across the interviews, highlighting their impact on the individuals’ perception of QoL. The multiformity on the parameters defining partner relationships after SCI, presents an opportunity for further future investigations.

Physical problems and long-term complications of SCI such as pain and incontinence have a negative fallout on QoL of individuals with SCI [35–37]. Specifically, neuropathic pain has been identified as a negative contributing factor for QoL after SCI [38–40]. In an Italian study on 385 individuals with SCI, Stampacchia et al reported that 72% of the cohort suffered from pain and specifically neuropathic pain was reported in 48% of the cases [41]. Similarly, we have reported on the occurrence of neuropathic pain at 47% at follow-up in a cohort of 194 individuals with cervical SCI [42]. These findings consistently illustrate the magnitude of this difficult to treat condition. Furthermore, the only domain in this study that rendered responses with a score below five (Question 1 – Physical well-being), concerned the role of pain as a decisive component in the subjective QoL (Table 2). The multiple mentions of neuropathic pain, across the interviews, puts on center stage its crucial role as a QoL determinant and emphasizes the need for continued efforts to develop more effective treatments.

### Strengths and limitations

The qualitative descriptive design effectively met the study’s aims. Trustworthiness was ensured through thorough analysis by experienced interviewers, clear participant descriptions, and a consistent analytic process. Rich data and clinical expertise supported credibility and transferability. Dependability was strengthened by standardized interviews conducted in a short timeframe [43]. Nonetheless, the study has some limitations. The voluntary participation inclusion strategy introduces a degree of self-selection bias. However, a saturation effect was observed towards the end of the study, where subsequent interviews provided no or only very limited additional data. This can be taken to suggests that the data collected accurately reflect the available study population. The uniform inclusion of patients in a nation-wide and state-funded spinal cord injury follow-up program ensures that socioeconomic or geographic differences do not affect access to health care. However, patients who do well without intense follow-up may not have been available for inclusion to the same degree as more severely affected patients. The findings reflect the views from only one outpatient clinic, exclude individuals with severe cognitive impairments, and may have lost nuances in translation from Swedish to English. While the study is relevant to the neurological challenges and needs of all SCI injury patients, the experiences related to the provided healthcare reflect that of a high-income western society.

## Conclusion

The participants in this study turned the spotlight to independence, social interconnection, and management of SCI-related physical complications as crucial components of their QoL. Notably, they reported that successful surgical interventions, including device implantations, had a meaningful and positive impact on their overall well-being. In addition, the community plays a pivotal role in enabling individuals with SCI to live a meaningful life beyond disability and in empowering them in the pursuit of their personal endeavors and aspirations. Personal assistance services for individuals with SCI play a paramount role on independence and participation in society. Services provided by the community are often essential to the individual’s pursuit of an independent and purposeful life and for the promotion of an inclusive environment that prevents social isolation.

## Data Availability

Data are available from the corresponding author upon reasonable request.
